# The in vivo location of edge-wear in hip arthroplasties: combining pre-revision 3D CT imaging with retrieval analysis

**DOI:** 10.1302/2046-3758.1010.BJR-2021-0132.R1

**Published:** 2021-10-04

**Authors:** Sean Bergiers, Harry Hothi, Johann Henckel, Anna Di Laura, Martín Belzunce, John Skinner, Alister Hart

**Affiliations:** 1 Institute of Orthopaedics and Musculoskeletal Science, University College London, London, UK; 2 Royal National Orthopaedic Hospital NHS Trust, Stanmore, UK

**Keywords:** Wear, Retrieval analysis, CT, hip arthroplasties, hip joints, hip implants, CT scans, acetabular component positioning, metal-on-metal hip arthroplasties, acetabular cartilage, acetabular components, acetabular cups, Subluxation

## Abstract

**Aims:**

Acetabular edge-loading was a cause of increased wear rates in metal-on-metal hip arthroplasties, ultimately contributing to their failure. Although such wear patterns have been regularly reported in retrieval analyses, this study aimed to determine their in vivo location and investigate their relationship with acetabular component positioning.

**Methods:**

3D CT imaging was combined with a recently validated method of mapping bearing surface wear in retrieved hip implants. The asymmetrical stabilizing fins of Birmingham hip replacements (BHRs) allowed the co-registration of their acetabular wear maps and their computational models, segmented from CT scans. The in vivo location of edge-wear was measured within a standardized coordinate system, defined using the anterior pelvic plane.

**Results:**

Edge-wear was found predominantly along the superior acetabular edge in all cases, while its median location was 8° (interquartile range (IQR) -59° to 25°) within the anterosuperior quadrant. The deepest point of these scars had a median location of 16° (IQR -58° to 26°), which was statistically comparable to their centres (p = 0.496). Edge-wear was in closer proximity to the superior apex of the cups with greater angles of acetabular inclination, while a greater degree of anteversion influenced a more anteriorly centred scar.

**Conclusion:**

The anterosuperior location of edge-wear was comparable to the degradation patterns observed in acetabular cartilage, supporting previous findings that hip joint forces are directed anteriorly during a greater portion of walking gait. The further application of this novel method could improve the current definition of optimal and safe acetabular component positioning.

Cite this article: *Bone Joint Res* 2021;10(10):639–649.

## Article focus

- Determining the in vivo location of edge-wear in hip arthroplasties, by combining pre-revision 3D CT imaging with retrieval analysis.

## Key messages

- Acetabular edge-wear was predominantly centred about an anterosuperior position in vivo, consistent with anteriorly directed hip joint forces and previously reported cartilage degradation patterns.
- A greater degree of acetabular inclination resulted in edge-wear scars centred about the superior apex of the cup.
- Acetabular anteversion was observed to influence the anteroposterior location of edge-wear scars.

## Strengths and limitations

- A novel approach to locating the in vivo location of acetabular edge-wear is introduced.
- The application of a newly validated method of quantifying and mapping material loss from the bearing surface of hip arthroplasties.
- This study was limited by its small sample size, which restricted the statistical evaluation of observed trends.

## Introduction

Since mechanical wear debris was identified as a contributor to hip arthroplasty failure, the tribological performance of these implants has been extensively analyzed. Nowhere has such material loss had a greater impact than on patients with metal-on-metal (MOM) hips. The release of cobalt and chromium particles was found to cause inflammation and necrosis in periprosthetic tissue,^1,2^ while the extent of its systemic effects remains unknown. Retrieval studies have been able to accurately quantify the volume of material loss from these implants, facilitating the identification of surgeon, implant, and patient (SIP) factors that contributed to their failure.^3,4^

The precisely polished bearing surfaces of hip arthroplasties are designed to facilitate low friction articulation. Under optimal conditions, load is transferred through the centre of both acetabular components and femoral head components during function.^5^ Certain SIP factors, however, are thought to cause this contact patch to move towards the interior edge of the acetabular component.^6,7^ This is often referred to as edge-loading and can lead to elevated levels of material loss.

Although wear is regularly identified at the edge of acetabular cups during retrieval analysis,^8^ its location in vivo has yet to be accurately determined. A previous study has demonstrated the feasibility of such measurements; however, their findings were absent of any clear trends.^9^ It has been hypothesized that acetabular component malpositioning increases the prevalence of edge-wear, particularly with excessive angles of inclination. Under these circumstances, the contact patch is thought to be in closer proximity to the acetabular edge. Nevertheless, along with the impact of anteversion, this is largely debated due to the contrasting findings of both clinical and retrieval studies.

Consequently, the overarching aim of this study was to determine the in vivo location of acetabular edge-wear, by combining pre-revision 3D CT images with retrieval analysis. This could provide a better understanding of the tribological performance of these bearings and the load distribution during function. This research also intended to identify whether acetabular component positioning influences the location of edge-wear.

## Methods

### Materials

**Fig. 1:**
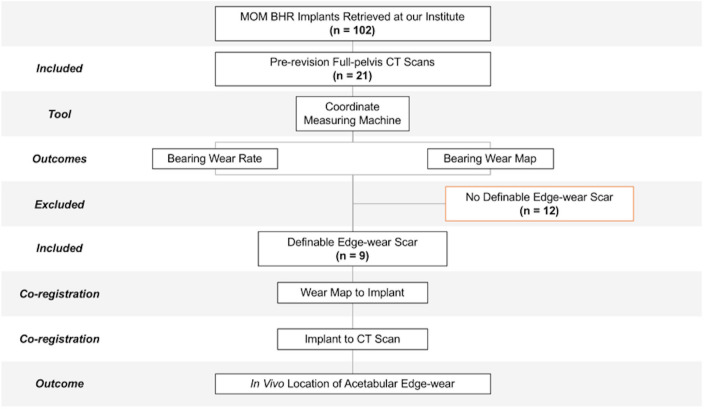
A schematic diagram of the study design. This includes the adoption of an exclusion criteria and the steps followed to determine the in vivo location of acetabular edge-wear in Birmingham Hip Replacements (BHRs). MOM, metal-on-metal.

The MOM Birmingham Hip Replacement (BHR; Smith & Nephew, UK) was considered the most appropriate implant for this research, due to the asymmetric stabilizing fins found at the backside of its cobalt-chromium-molybdenum (CoCrMo) acetabular component. As these design features would be visible in pre-revision 3D CT scans, the orientation of the implant could be defined within the patient. In total, 102 retrieved BHRs were revised by two of the authors (JS and AH). Prior to revision surgery, a full-pelvis 3D CT scan was performed in 21 of these cases as part of their routine clinical follow-up, making them potentially eligible for this study (Figure 1).

These hips were revised due to an adverse reaction to metal debris (ARMD; n = 9), unexplained pain (n = 9), and component loosening (n = 3), after a mean time of 89.1 months (15 to 177). Their mean cup size was 53.7 mm outer diameter (48 to 62), while their nominal diametrical clearance and arc of coverage angle were 200 μm and 158° to 166°, respectively.^10^ They were explanted from 14 females and seven males, who had a mean age of 56.7 years at implantation (35.4 to 73.8).

A further inclusion criterion of this study was the identification of a clearly defined primary edge-wear scar on their acetabular bearing surface, which would be determined through tribological metrology.

### Volumetric wear measurements of BHR acetabular cups

A Micura coordinate measuring machine (Carl Zeiss Ltd, UK) was used to capture the geometry of the acetabular bearing surface of each retrieved BHR implant in the form of a point cloud. The adopted scanning strategy was optimized for each component size, conforming to the guidelines of both International Organization for Standardization (ISO) and ASTM standards.^11,12^ The first arc of each scan was consistently aligned to the same stabilizing fin, which was confirmed by further data points recorded at the apex of this fin. A previously validated, automated software solution was used to analyze the acquired point clouds,^13^ quantifying the volume of material loss from each BHR acetabular component. 3D wear maps were also generated, allowing the identification of a primary edge-wear scar. The limits, centre, and deepest point of this wear scar were then determined from these maps.

### Measurement of acetabular component position

**Fig. 2:**
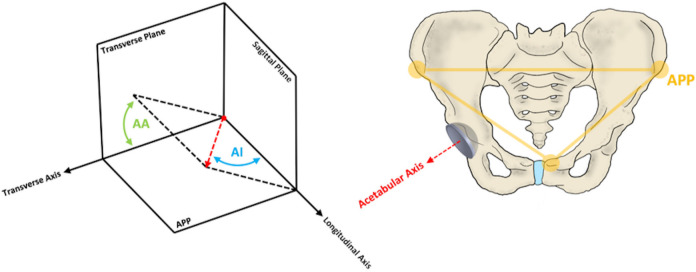
A schematic diagram demonstrating the definition of the anterior pelvic plane (APP) and the measurement of anatomical acetabular cup inclination (AI) and anteversion (AA). The APP forms the coronal plane, from which the sagittal and transverse planes can be defined. The acetabular axis intersects the centre of the cup and is perpendicular to the cup rim plane.

A bespoke software solution (Robin’s 3D; Robin Richards, UK) was adopted to measure the position of each BHR acetabular component, which automatically generated high-resolution 3D computational pelvis and implant models from the CT scans. The anterior pelvic plane (APP) was used to form a standardized coordinate system within each patient, defined using both anterosuperior iliac spines and the anterior surface of the pubic symphysis (Figure 2). This allowed angles of inclination and anteversion to be subsequently measured.

### Co-registration of acetabular wear maps to CAD models

**Fig. 3:**
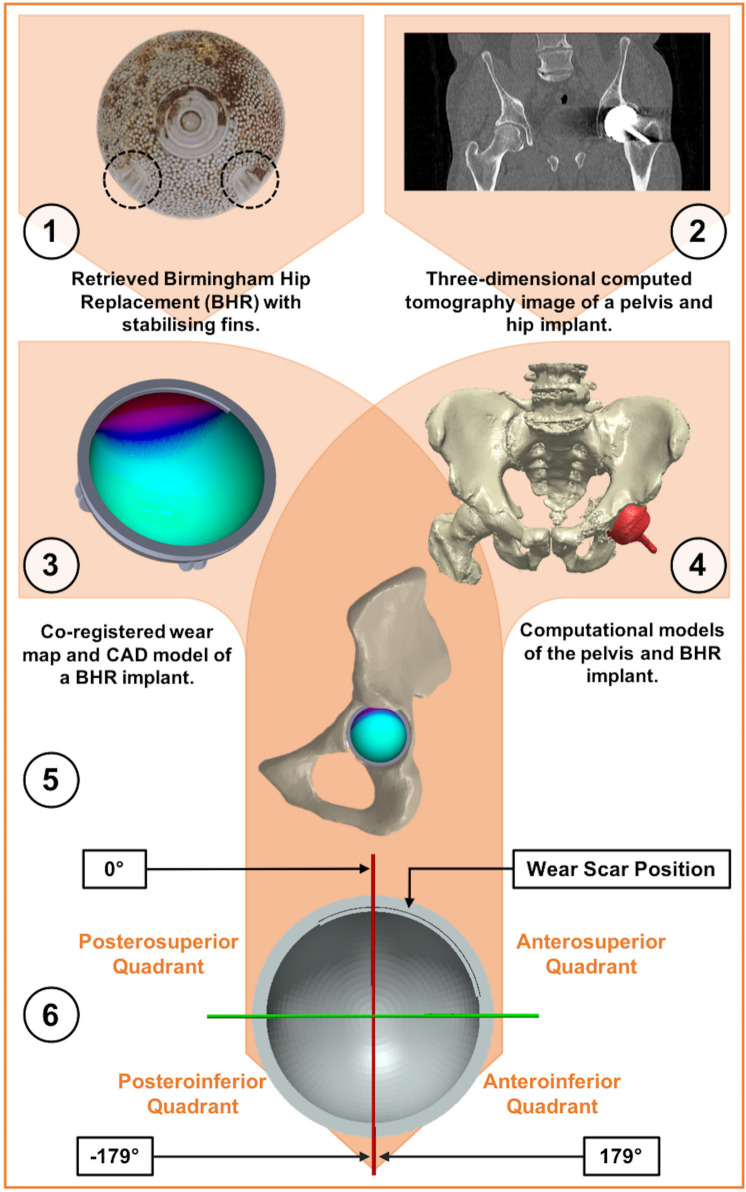
A schematic diagram depicting the process of locating acetabular edge-wear in vivo. 1) A macroscopic image of a retrieved Birmingham acetabular component (backside), where its stabilizing fins have been circled. 2) A single slice of a pelvic CT scan, which includes a cobalt-chromium-molybdenum (CoCrMo) Birmingham hip replacement (BHR) metal implant. 3) A wear map co-registered to a BHR (52 mm) computer-aided design (CAD) model. 4) Computational models of the pelvis and BHR implant, segmented from a 3D CT scan. 5) A BHR implant CAD model and wear map, co-registered to the computational model of the actual implant within the pelvis. 6) A perpendicular view of the acetabular cup and its measurement axis, where the Cup-APP (CAPP) plane defines the 0° and 180° positions. Anterior and posterior locations of edge-wear were defined by positive and negative angles, respectively.

A 3D CAD model of each acetabular BHR component was produced using SolidWorks (Dassault Systèmes SolidWorks Corporation, USA), which was informed by dimensional CMM measurements. The previously generated wear maps could be co-registered to these CAD models, as their orientation had been established relative to the stabilizing fins. A projection of each wear map was manipulated on the articulating surface of their CAD model to achieve this alignment. The location of the primary edge-wear scar was marked by extruding its expanse from the rim of the model (Figure 3).

### Co-registration of CAD models to 3D CT images

First, a pre-revision, full pelvis CT scan of each patient was imported into Synopsys Simpleware ScanIP (Synopsys, USA) as an anonymized stack of Digital Imaging and Communications in Medicine (DICOM) images. These were compiled to form a 3D image composed of voxels, each with their own grayscale intensity. Material density dictated X-ray attenuation during CT imaging and consequently the grayscale values of these voxels, allowing computational models of the patient’s pelvis (bone) and BHR hip implant (CoCrMo) to be segmented from other materials (Figure 3). This was achieved using a semi-automated process called ‘thresholding’, which involved defining a range of grayscale values representative of each material. A grayscale range of approximately 130 to 1,200 was used to isolate bone from each image stack, while the BHR implants were segmented using a range of grayscale values often above 1,800. The authors’ discretion was required as the grayscale varied in magnitude, based on scanning parameters. Computational post-processing tools were used sparingly to remove metal artifacts from the models, without impacting anatomical dimensions. An automated registration function was used to co-register the BHR CAD model to the actual acetabular component segmented from the CT scan (Figure 3). Manual input was required to refine the resulting fit, which involved selecting landmarks such as the rim and stabilizing fins to inform the alignment.

### Measurement of the in vivo location of edge-wear

**Fig. 4:**
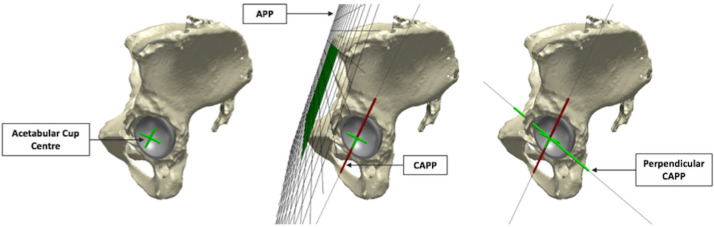
Computational models of a pelvis and Birmingham hip replacement (BHR) implant, generated within Simpleware ScanIP. The "For Review Only" standardized reference system defined to measure the in vivo location of edge-wear is illustrated (Cup-APP (CAPP)), which is parallel to the anterior pelvic plane (APP) and intersects the centre of the cup opening.

The in vivo location of acetabular edge-wear was determined within the Simpleware ScanIP software. As with the measurements of component positioning, the APP was used as a standardized reference between patients. A plane termed the Cup-APP (CAPP) was defined, which was parallel to the APP and intersected the centre of the BHR cup opening (Figure 4). The two points at which the CAPP intersected the acetabular component rim were used to define the 0° and 180° limits of the measurement system, as seen in Figure 3. Vertical and horizontal axes were formed from these points, within the acetabular rim plane, dividing the articulating surface into four quadrants. As a result of its relationship to the APP, 0° was considered representative of the vertical standing pelvic position.^14,15^ Component position was then neutralized, achieving a perpendicular view of the acetabular component rim, with the vertical axis positioned appropriately. The angle between both limits of the edge-wear scar were measured, with respect to the vertical axis. As both right- and left-sided implants were included in this study, the anterior and posterior halves of the acetabular rim were represented by positive and negative angles, respectively (Figure 3).

To visualize the in vivo location of wear with respect to the pelvic anatomy, the implant and its hemipelvis were exported from Simpleware ScanIP. This was re-imported into SolidWorks, where the original CAD model and co-registered wear map could be re-aligned to the stabilizing fins (Figure 3).

### Statistical analysis

All steps associated with the presented method of locating acetabular edge-wear in each implant were repeated by a single observer (SB), with the intention of evaluating measurement repeatability. Bland–Altman plots were generated to compare the outcome of these analyses, obtaining the mean error between measurements, in addition to upper and lower 95% limits of agreement. The locations of the centre and deepest points of the edge-wear scars were compared using a Wilcoxon paired *t*-test, while a Mann–Whitney U test was performed to compare the wear rates of edge worn and non-edge worn BHR implants.

## Results

**Fig. 5:**
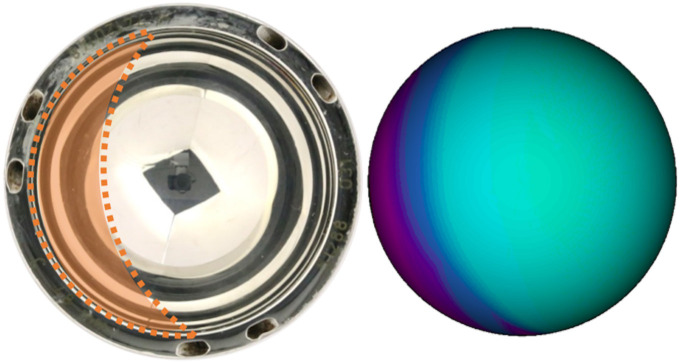
A macroscopic image and wear map of the bearing surface of a Birmingham hip replacement (BHR) acetabular component. The edge-wear scar is highlighted in both representations of the implant.

A median wear rate of 0.41 mm^3^/year (interquartile range (IQR) 0.11 to 5.65) was measured from the 21 BHR acetabular components. Nine of these implants had an identifiable primary edge-wear scar, which shared a common crescent shape and were bound by the articulating surface edge throughout their expanse (Figure 5). In accordance with the inclusion criteria, the remaining 12 cups were excluded from the study due to their evenly distributed wear patterns and would not undergo further analysis.

The edge-worn implants had been positioned with a median value of inclination and anteversion of 60° (IQR 53° to 67°) and 39° (IQR 19° to 48°), respectively. According to Lewinnek’s safe zone,^16^ these were collectively malpositioned, with all nine implants falling outside this zone. Edge-worn BHR acetabular components were found to have a significantly greater (p = 0.007, Mann–Whitney U test) median bearing surface wear rate of 4.52 mm^3^/year (IQR 1.16 to 19.28), compared to the non-edge-worn components that had a median wear rate of 0.14 mm^3^/year (IQR 0.04 to 0.50).

**Fig. 6:**
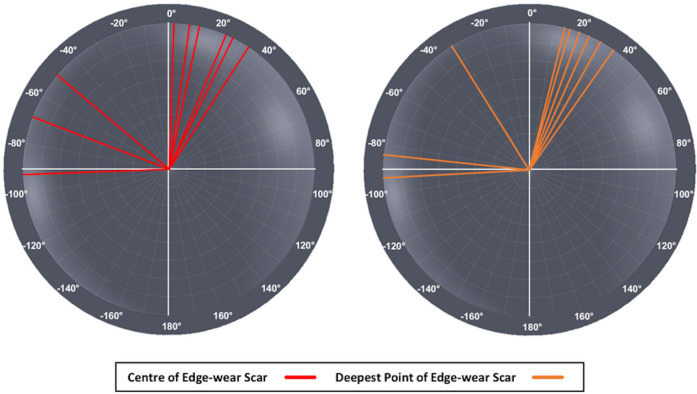
The in vivo location of the centre (red) and deepest point (orange) of each acetabular edge-wear scar.

**Fig. 7:**
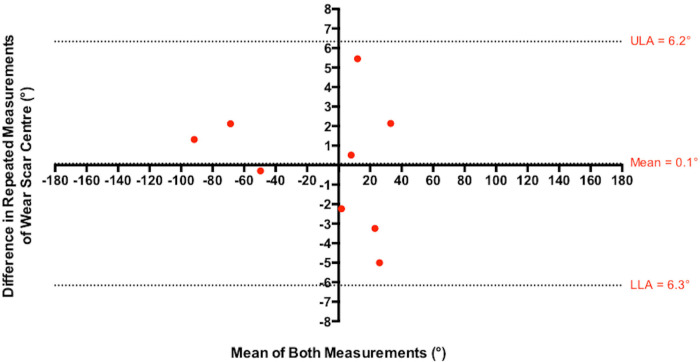
A Bland–Altman plot comparing the repeated measurements of the in vivo location of each acetabular edge-wear scar centre, performed by a single observer. The mean error is presented, in addition to the upper (ULA) and lower (LLA) 95% limits of agreement.

The centre of these edge-wear scars had a median in vivo location of 8° (IQR -59° to 25°; Figure 6). Comparing both repetitions of this analysis, the Bland–Altman plot displayed in Figure 7 found that these measurements had a mean error of 0.1° (standard deviation (SD) 3.2°), and upper and lower 95% limits of agreement of 6.2° and -6.3°, respectively. The deepest point of wear had a median in vivo location of 16° (IQR -58° to 26°), which was found to be statistically comparable to their centres (p = 0.496, Wilcoxon paired *t*-test). Both sets of measurements suggest that edge-wear was most prevalent in the superior anterior quadrant of the BHR acetabular components. Disregarding wear coverage extending towards the pole, the distribution of edge-wear was most prevalent on the superior edge of the acetabular bearing surface, despite the median breadth of edge-wear being 154° (IQR 111° to 164°).

**Fig. 8:**
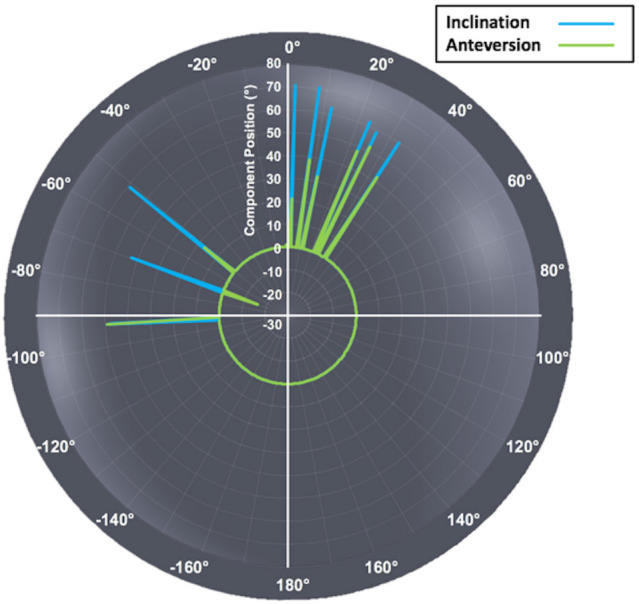
The relationship between the in vivo location of acetabular edge-wear and acetabular component positioning, which is represented by angles of inclination (blue) and anteversion (green).

Through observations alone, higher volumetric wear rates were associated with more anteriorly centred edge-wear scars. With respect to acetabular component position, edge-wear scar centres were found to tend towards the apex of the cup when positioned with larger angles of inclination, while a greater degree of anteversion was found to result in the anterior migration of edge-wear (Figure 8).

## Discussion

The combination of pre-revision 3D CT images and retrieval analysis allowed edge-wear to be located along the superior bearing surface edge of all BHR acetabular components that demonstrated this wear pattern. The centre and deepest points of these wear scars were found to be more prevalent in the anterosuperior quadrant of these cups, suggesting that load was most frequently transferred through this region of their bearing surfaces. Edge-wear was also found to span a large portion of the superior acetabular edge, which could be attributed to the range of hip motion, from flexion to extension, during day-to-day activity. It must be acknowledged that this study selectively analyzed edge-worn hips and not all BHRs presented this wear pattern. The novel approach adopted in this study benefited from a newly validated method of quantifying and mapping bearing surface wear, allowing the boundary of each scar to be clearly defined. An evaluation of its repeatability determined that edge-wear could be located within mean error of 0.1° (SD 3.2°), while the maximum measurement error (6.3°) would not alter the acetabular quadrant within which it was located.

**Fig. 9:**
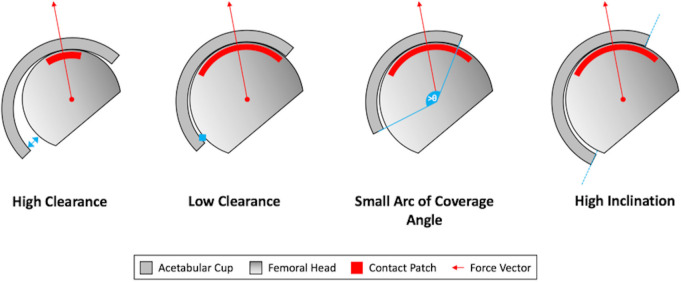
A schematic diagram depicting the influence of clearance, arc of coverage angle, and acetabular inclination on the location of the bearing contact patch location.

Although edge-wear is commonly identified on the acetabular bearing surface of retrieved MOM hip arthroplasties, the location at which it occurs in vivo had not been previously identified. Multiple SIP factors are understood to reduce the proximity between the contact patch of these bearings and the acetabular rim, which can include small clearances, small arc of coverage angles, and high inclination angles (Figure 9). Although all three of these contributors to edge-loading are thought to cause superior acetabular edge-wear in hip arthroplasties, this has not been previously confirmed by retrieval findings. While this may be a logical assumption considering the hip joint biomechanics, what has been more difficult to deduce is the anteroposterior (AP) distribution of edge-wear from a sagittal perspective of the pelvis.

In agreement with the findings of the present study, musculoskeletal modelling has previously demonstrated that hip joint forces are directed anteriorly during a greater portion of walking gait, accounting for nearly 40% of the stance phase.^17,18^ This is the predominant weight-bearing stage of the average walking cycle, which terminates when maximum extension is achieved and the toe push-off is performed. Contrastingly, instances of posteriorly directed hip joint force have only been detected during the heel-strike.^17,19^ Lewis et al^20^ found that the amount of anteriorly directed hip joint force was also positively correlated with the degree of extension performed during the walking gait cycle.^21^ This would suggest that the majority of individuals included in this study were using the allowable range of motion of their hip implants.

Comparable to the in vivo location of edge-wear, acetabular cartilage degradation is also found to be most severe at the anterosuperior portion of native hip joints. These patterns have been observed during interoperative and MRI assessments of osteoarthritic joints,^22-24^ and more recently confirmed using delayed gadolinium-enhanced MRI (dGEMRIC),^25-29^ which quantitatively measures contrast agent penetration into the cartilage tissue.^30^ Again consistent with the present investigation, the presence of cartilage damage is also found to span across the superior portion of the acetabulum, including posterior regions. Research into cartilage degradation patterns have primarily assessed diseased joints;^31,32^ nevertheless, similar patterns have been observed in asymptomatic individuals.^33^ Acetabular cartilage not only allows smooth hip movement, but also performs as a ‘shock-absorber’ that facilitates a more even distribution of forces. This property of cartilaginous connective tissue must be acknowledged when making such analogies with CoCrMo acetabular cups, as it certainly influences the resulting degradation patterns. Moreover, the progression of OA is more complex than mere ‘wear and tear’ of cartilage alone, but is rather a disease of the entire joint that can be triggered at a cellular level.^25,34^

Evidence acquired through instrumented hip implants further supports the results of this study. Such devices rely on embedded sensors to provide in vivo force data.^35,36^ Hodge et al^37^ found that the maximum pressure applied to the acetabulum during the stance phase of gait was located in an anterosuperior position, comparable to the median location of edge-wear scar centres measured in this study (8°; IQR -59° to 25°). Furthermore, the location of this maximum pressure was found to move to the posterior aspect of the acetabulum when rising from a chair.^37^

**Fig. 10:**
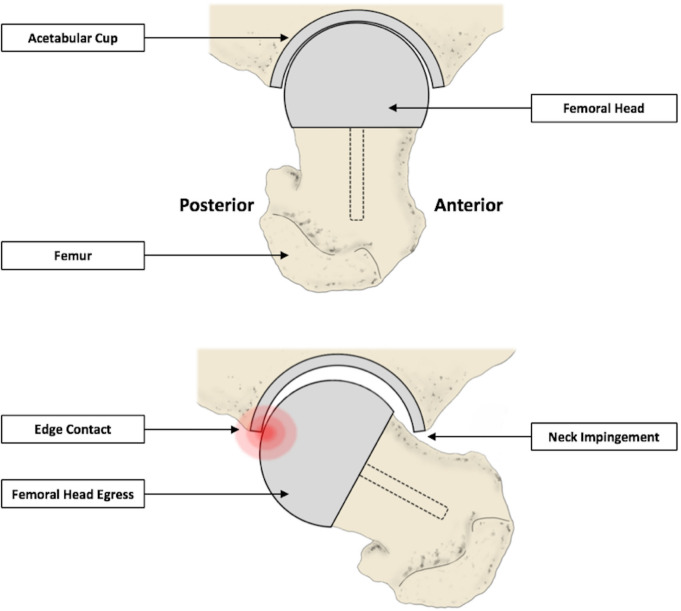
A schematic diagram representing posterior edge contact, caused by instances of anterior impingement. Edge-wear can occur at the egress site, as the femoral head is levered out of the acetabular cup.

Subluxation may further explain these instances of posterosuperior edge-wear, as it has been previously associated with increased stresses at the surface of bearings and irregular wear patterns.^38^ This refers to the brief dislocation and recoupling of the femoral head and acetabular components of hip implants.^39^ Subluxation is particularly prevalent in hip arthroplasties such as the BHR, as it has a large nominal clearance (200 μm) relative to other MOM bearings.^40^ Through finite element analysis, Elkins et al^41^ reported the vulnerability of the posterior acetabular component rim to head egress, resulting in greater applied stresses at this region. This can occur during flexion and the impingement of the femoral neck on the opposing acetabular edge (Figure 10). As the three examples of posteriorly centred edge-wear were relatively low-wearing, subluxation scars may have dominated their wear profiles.

This study builds upon our previous investigation into the in vivo location of acetabular wear.^9^ In contrast to the present research, this previous study reported a considerable amount of variability in its findings. Both superior and inferior regions of wear were observed, which opposed the theory of a superiorly located contact patch during edge-loading. As this study was not solely focused on edge-wear, such results may be understandable due to the more evenly distributed wear patterns found in low-wearing acetabular components.^6,38^ This can make identifying primary wear scars more difficult, as isolated instances of edge-wear can appear more prominent in these cases. Such wear patterns can be caused by impingement due to extreme gait or movement, as well as subluxation or even severe three-body abrasion. The possibility that similar areas of damage were located in the previous study may explain its identification of inferiorly positioned edge-wear.^9^

Interestingly, the acetabular position of the BHRs included in this study appeared to influence the in vivo location of their edge-wear. Implants with a greater degree of inclination were more likely to have an edge-wear scar centred about the superior apex of the cup. Their angle of anteversion seemed to dictate the AP location of edge-wear, as the scars were found to be centred more anteriorly in implants with increased anteversion. Although a greater sample of implants would be required to confirm these observations, they do conform with the hypothesis that the cup-head contact patch moves superiorly towards the acetabular edge as the inclination increases from the optimal range.^42^ Similarly, they are consistent with the previously discussed contribution of subluxation, as increased anteversion can lead to a reduced probability of anterior impingement and posterior edge contact.^41^

The exception to these theories was the greatly anteverted BHR implant that had a wear scar centred at -90° (posterior). This may be explained, however, by posterior impingement of the femoral neck on the cup rim during extension.^43,44^ The prominence of the resulting scar would be understandable considering that this was the lowest wearing BHR to display a clear primary edge-wear scar. Human walking gait is also known to vary considerably between individuals and can be further affected by hip disease,^45,46^ further contributing to such irregular wear patterns.

A clear relationship between the performance of MOM hip arthroplasties and their inclination angles has not always been reflected in clinical and retrieval findings.^47-49^ There is even less evidence to confirm the impact of anteversion on either wear performance or clinical outcomes.^38,47^ The optimal degree of anteversion of an implant is considered the point at which the cup-head contact patch is located centrally about the posterior-anterior axis of the sagittal plane.^5^ If the in vivo location of wear is considered representative of this contact patch, the findings of this study would suggest that this optimal position would be achieved through approximately 20° of anteversion. Although consistent with the safe zone suggested by Lewinnek et al,^16^ this guide is known to have limitations, such as its disregard of functional position during daily activities.^50,51^ Its vertical position during function has also been debated, with a few degrees of error being proposed in a percentage of the population.^14^

Accurately positioning the acetabular component of hip arthroplasties is challenging, especially considering that the range of optimal angles remains unclear.^43,52^ Implant design can also dictate the surgical margin for error before edge-loading can become an issue. MOM hip arthroplasties were ultimately limited by the fact that even small amounts of metal debris had an adverse effect on periprosthetic tissue,^53^ reducing the size of this optimal positioning window. The navigation systems and robotic interventions now used to enhance surgical accuracy may have improved the clinical outcome observed in these MOM hips; however, it is unlikely that failure would have been avoided due to the multitude of other contributing factors. The native position of the acetabulum can also be influential, raising the question of whether hip implants should replicate the natural joint or augment its mechanics to achieve optimal conditions for reduced wear and improved performance.

It must be acknowledged that the primary limitation of this study was its small sample size, restricting the determination of statistical significance in the observed trends. This can be attributed to the scarcity of appropriate pre-revision CT imaging and the low prevalence of edge-wear in BHR implants. Nevertheless, the specific design of the BHR was crucial to this investigation, enabling the definition of their in vivo orientation. Although the strict inclusion criteria adopted in this study essentially excluded well-positioned implants, it avoided any uncertainty regarding the dominant wear scar. Gait analysis would complement future investigations of the in vivo location of edge-wear and inform the interpretation of their findings, in addition to the measurement of femoral positioning, activity levels, body mass, and pelvic tilt.

In conclusion, acetabular edge-wear was found to be predominantly centred about an anterosuperior location in vivo, in agreement with previous reports of hip joint forces being directed anteriorly during a greater portion of walking gait. As edge-wear was consistently located at the superior acetabular edge, it also supports the contribution of clearance, arc of coverage angle, and inclination to instances of edge-loading in MOM hip arthroplasties as previously theorized. For the first time, retrieval evidence was found to suggest the influence of acetabular anteversion on the AP location of the bearing contact patch and on edge-wear. Further adoption of this novel method could provide an insight into the distribution of load through hip arthroplasties and improve the current definition of the optimal and safe zones for acetabular component positioning.
